# Rare disease-based scientific annotation knowledge graph

**DOI:** 10.3389/frai.2022.932665

**Published:** 2022-08-11

**Authors:** Qian Zhu, Chunxu Qu, Ruizheng Liu, Gunjan Vatas, Andrew Clough, Ðắc-Trung Nguyễn, Eric Sid, Ewy Mathé, Yanji Xu

**Affiliations:** ^1^Division of Pre-clinical Innovation, National Center for Advancing Translational Sciences, Rockville, MD, United States; ^2^Division of Rare Diseases Research Innovation, National Center for Advancing Translational Sciences, Bethesda, MD, United States; ^3^GMG ArcData, LLC, Washington, DC, United States; ^4^Data Decode, LLC, Washington, DC, United States; ^5^Digital R&D Solutions, Pfizer, New York, NY, United States

**Keywords:** scientific annotations, natural language processing, knowledge graph, rare disease (RD), PubMed

## Abstract

Rare diseases (RDs) are naturally associated with a low prevalence rate, which raises a big challenge due to there being less data available for supporting preclinical and clinical studies. There has been a vast improvement in our understanding of RD, largely owing to advanced big data analytic approaches in genetics/genomics. Consequently, a large volume of RD-related publications has been accumulated in recent years, which offers opportunities to utilize these publications for accessing the full spectrum of the scientific research and supporting further investigation in RD. In this study, we systematically analyzed, semantically annotated, and scientifically categorized RD-related PubMed articles, and integrated those semantic annotations in a knowledge graph (KG), which is hosted in Neo4j based on a predefined data model. With the successful demonstration of scientific contribution in RD *via* the case studies performed by exploring this KG, we propose to extend the current effort by expanding more RD-related publications and more other types of resources as a next step.

## Introduction

**Figure 1:**
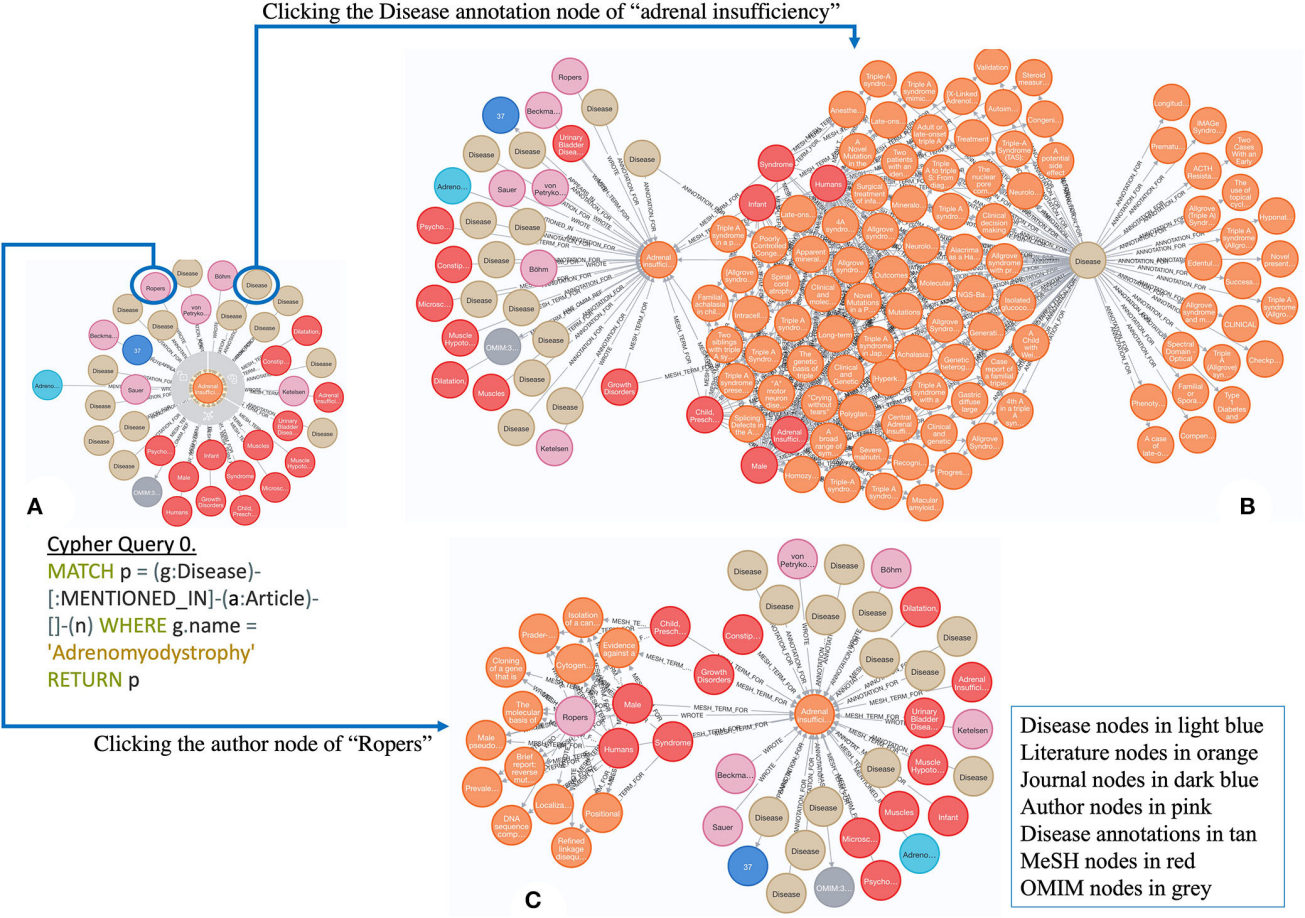
An example of exploring Adrenomyodystrophy-associated literature [**A**: a graph containing semantic annotations generated from the original paper for Adrenomyodystrophy; **B**: an expanded graph from **A** *via* one disease node of “adrenal insufficiency”; **C**: an expanded graph from A *via* one author node of “Ropers.” The attached Cypher Query 0 was applied to generate **A**. **(B,C)** can be generated by clicking the nodes circled in **A**, respectively].

There are ~7,000 rare diseases (RDs), which affect nearly 30 million Americans combined. Notably, 80% of patients are affected by ~350 RDs, and the other 20% are affected by the rest of the 7,000 RDs^1^. Most RDs are associated with low prevalence rate (Shourick et al., 2021), some being exceptionally rare. For instance, Adrenomyodystrophy (GARD: 0000562) is an extremely rare genetic endocrine disease characterized by primary adrenal insufficiency and hepatic steatosis. There has been no further study about this disease since 1982 (Von Petrykowski et al., 1982), which causes significant challenges to better understand its underlying mechanism for subsequent management. In this case, scientific evidence derived or extended from this literature would be useful for supporting its further research. Figure 1 illustrates an example of extending annotations derived from its original publication of Adrenomyodystrophy (Von Petrykowski et al., 1982) to identify more related articles for further investigation. To be specific, Figure 1A includes 14 disease concepts, 14 MeSH terms, 1 OMIM term, and a list of author nodes annotated from Adrenomyodystrophy (in light blue)-associated literature (Von Petrykowski et al., 1982) (in orange). These different types of annotations as entry points enable to discover additional information about Adrenomyodystrophy, which is shown in Figures 1B,C by expanding the knowledge graph (KG) in 1-A *via* one disease annotation node of “adrenal insufficiency” and one author node of “Ropers,” respectively. Thus, we have greater scientific space to investigate more about Adrenomyodystrophy, which is our motivation to generate and integrate various types of annotations from those publications into a KG for advancing rare disease research.

Many resources have been used to integrate and analyze scientific literature for supporting rare disease research. The Encyclopedia of Rare Disease Annotations for Precision Medicine (eRAM) (Jia et al., 2017) was built by mining nearly 10 million scientific publications and electronic medical records, and integrating various data from the existing recognized databases, including Unified Medical Language System (UMLS), Human Phenotype Ontology (HPO) (Robinson and Mundlos, 2010; Köhler et al., 2017), and Orphanet (Weinreich et al., 2008). Online Mendelian Inheritance in Man (OMIM™) (Amberger et al., 2015) contains comprehensive information on all known Mendelian disorders from the literature. The Mondo Disease Ontology (Mondo) (Vasilevsky et al., 2020) aims to harmonize disease definitions across the world and includes all relevant publications as references. Although scientific publications have been intensively mined and integrated with these resources, none of them exposes derived scientific evidence explicitly in a semantic form, as shown in Figure 1, to support further investigation. In this project, we introduced a KG by semantically integrating and representing scientific evidence derived from RD-related publications for effective exploration.

Knowledge graphs have been widely adopted to manage big data in the biomedical domain, given its merits of computational capacity. Zhao et al. (2019) reported that hosting disease data as KG in Neo4j^2^ provide a more intuitive image analysis approach to assist with a treatment recommendation. Stark et al. (2017) presented a drug recommendation system based on a highly scalable native graph database in Neo4j. We previously developed a KG named NCATS GARD Knowledge Graph (NGKG) with more than 40 biomedical resources (Zhu et al., 2020). This KG has been applied as a data foundation to support various biomedical application development, including the Genetic and Rare Disease (GARD) program^3^. In this study, we retrieved the RD list and its relevant data from the NGKG, which can be accessed at https://disease.ncats.io/browser. Thereafter, we were able to annotate and integrate scientific annotations from RD-related literature in a KG for supporting scientific research, which is demonstrated in the “Case studies” section.

## Methods

**Figure 2:**
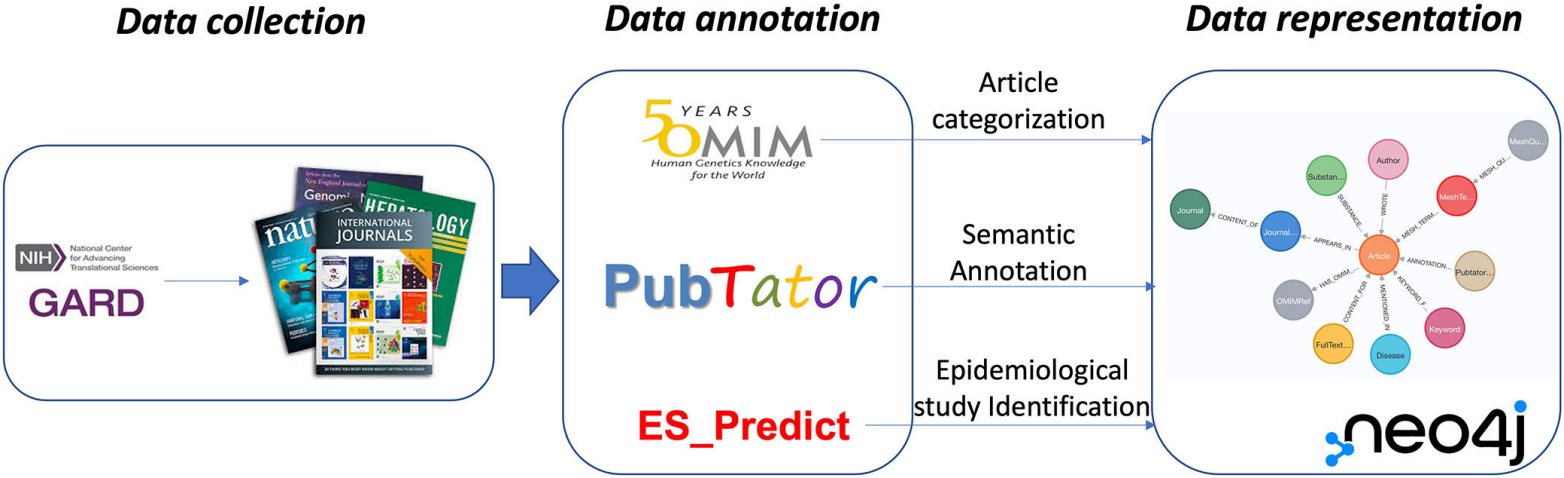
The study workflow.

In this study, we introduced an RD-centralized scientific annotation-based KG to organize and represent scientific evidence derived from PubMed literature for RD. Ultimately, it supports scientific research in RD. The workflow of this study is shown in Figure 2.

### RD-based publication collection

#### RD data preparation

As we mentioned, the NGKG contains RD-related data from GARD, and we retrieved 6,061 GARD RDs from the NGKG by executing the Cypher Query 1 with GARD IDs and GARD names as output. Then, GARD names were applied as input parameters to search for relevant PubMed articles.

Cypher Query 1.

MATCH     p     =     (d:DATA)-[:PAYLOAD]->(g:S_GARD)
WHERE d.is_rare = True
RETURN DISTINCT   d.gard_id  AS  GARD_ID, d.name
AS GARD_Name

#### Data collection from PubMed

We collected PubMed articles for those GARD diseases *via* two steps, namely, (1) for each individual RD, a maximum of 1,000 PMIDs with publication dates between 1 January 1900 and 31 August 2021, were retrieved by invoking the NCBI E-utilities API (Sayers, 2009) and (2) for each obtained PMID, PubMed abstract along with publication-related metadata, including publication title, keywords, publication types, MeSH terms^4^, and substances^5^, was collected from Europe PMC^6^.

#### Data collection from OMIM

OMIM (Amberger et al., 2015) is a comprehensive, authoritative compendium of human genes and genetic phenotypes curated by referencing different publications. To extract references for each individual RD from OMIM, we first obtained OMIM ID for each disease if applicable by executing the Cypher Query 2. Cypher Query 2 was mainly relied on exact and close match relationships (i.e., “R_exactMatch,” “R_equivalentClass,” and “R_closeMatch”) among Orphanet, MONDO, GARD, and OMIM curated by MONDO and Orphanet. With each OMID ID as an input, we were able to search the OMIM profile including references *via* OMIM API^7^. Since PubTator (Wei et al., 2013) only annotates PubMed articles (in the “Annotating articles with PubTator” section), only references indexed by PubMed were included in this study.

Cypher Query 2.

MATCH                        (o:S_ORDO_ORPHANET)-[:R_exact
Match|:R_equivalentClass]-(m:S_MONDO)-
[:R_exactMatch|:R_equivalentClass]-(n:S_GARD)<-
[:PAYLOAD]-(d:DATA)
WHERE d.is_rare=true WITH o,n,m,d
MATCH                         (o)-[e:R_exactMatch|R_closeMatch]-
(k:S_OMIM) <-[:PAYLOAD]-(h:DATA)
RETURN DISTINCT  d.gard_id  as  GARD_ID, d.name  as
GARD_Name, e.name as Orphanet_Match_Type, h.notation
as OMIM_ID, h.label as OMIM_Name
ORDER BY GARD_ID

### Semantic annotation on PubMed articles

Once we collected RD-related PubMed articles, we aimed at identifying and extracting the occurrences of biomedical concepts in those articles and representing them in a centralized KG to support scientific discovery. In this study, we performed three different approaches to annotate those articles, respectively.

#### Annotating articles with PubTator

To programmatically extract key concepts mentioned in article titles and abstracts, we employed PubTator (Wei et al., 2013), an NLP tool retrieving bio-concept annotations from biomedical articles in full text, to those collected PubMed articles from the above steps *via* the PubTator API^8^. Eight different types of biomedical concepts, i.e., diseases, mutations, species, genes, chemicals, cell lines, genus, and strain, have been extracted from the articles.

#### Categorizing articles with OMIM profiles

In the OMIM profile, cited references were embedded in different sections, such as clinical features, diagnosis, and clinical management. Such corresponding relationships between references and sections indicate the key contribution (i.e., category) of those articles. For instance, “Diagnosis of Wilson's disease: an experience over three decades” (PMID: 10673307) was cited in the “Clinical features” section for Wilson's disease (OMIM: 277900). It is clear that this publication aimed at reporting their experience over three decades in patients with Wilson's disease to illustrate the diverse patterns of clinical presentation (Gow et al., 2000). That is to say, we can group this article as a clinical feature-related category. Thus, while we extracted those references from each individual OMIM profile, we grouped them into different categories according to their corresponding sections.

#### Tagging articles with a predictive model

Epidemiology helps improve RD-related knowledge and facilitates policy decisions by considering the burden of RD in society. Given such incredible role of epidemiology plays in the field of RD, in our previous study (see text footnote 8), we developed a long short-term memory recurrent neural network-based epidemiology study predictor named ES_Predict to predict PubMed articles as epidemiology study. We applied the predictor over the collected PubMed articles to tag those predicted as epidemiology study.

### Data model development

**Figure 3:**
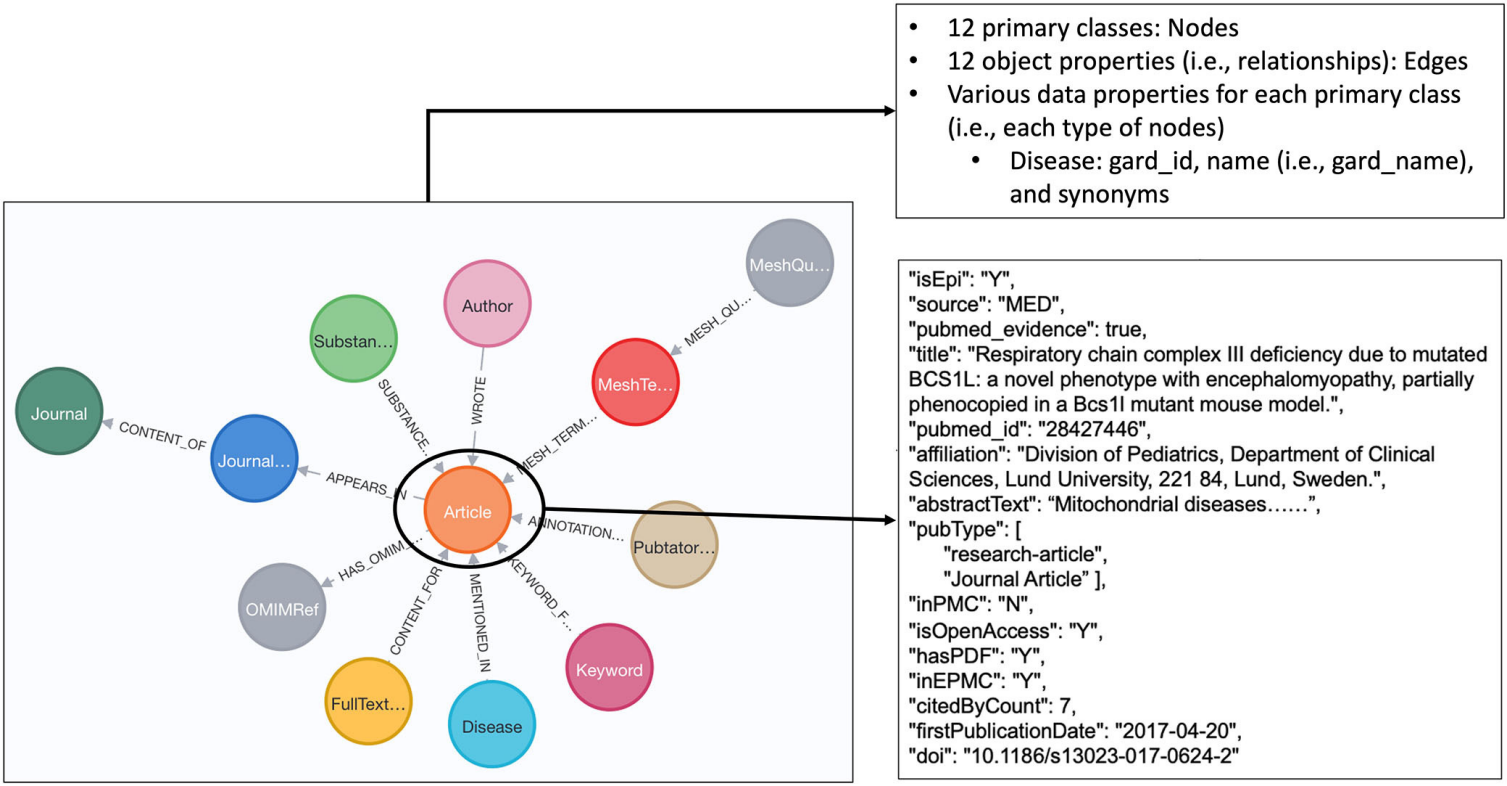
Data model and an example of information (data properties) about one article node.

We defined a data model to semantically capture and represent various types of data we extracted from the RD-related publications. As shown in Figure 3, twelve primary classes were defined accordingly, namely, Disease, Article, Author, Journal, Keywords, MeshTerm, PubTator Annotation, FullTextUrl, Substance, MeshQualifier, JournalInfo, and OMIMRef. Notably, OMIMRef was defined to capture OMIM information and category information from OMIM, which is described in the “Categorizing articles with OMIM profiles” section. The twelve object properties were defined to capture semantic relationships among those primary classes, shown as edges in Figure 3. A total of 56 data properties to depict descriptive information were defined and attached to each corresponding primary class. The semantic annotations generated from the “Semantic annotation on PubMed articles” section were attached to the Article nodes as individual data properties. To be specific, “isEpi” was defined to indicate whether this article is epidemiology study based on the prediction result from the ES_Predict, for instance, “isEpi”=Y, indicating the article is predicted as epidemiology study. Another data property of “pubmed_evidence” is defined to denote that the article is from PubMed and “omim_evidence” denotes that the article is from OMIM. “refInOMIM” indicates that the article is from PubMed (i.e., “pubmed_evidence=true”) and also referenced by OMIM.

### Knowledge graph development

Based on the data model we described earlier, we loaded extracted publication data along with their semantic annotations to a KG in Neo4j with the version 4.4.4. To be specific, different types of data have been loaded and represented by those twelve primary classes as nodes; object properties were applied to establish semantic connections between different nodes as edges, and data properties were attached to corresponding nodes as node properties. The KG is publicly assessable without login requirement at https://rdip2.ncats.io:7473.

## Results

A total of 1,362,819 PubMed articles were retrieved from PubMed and OMIM. Out of 45,484 references (with PubMed ID) from 4,203 OMIM profiles, 22,628 references were overlapped with articles from PubMed. There were 5,843 GARD RDs for which PubMed articles were retrieved, and there were 218 GARD diseases without PubMed articles.

### Results of semantic annotation

#### Pubtator annotations

There were 1,634,387 PubTator annotations generated from 1,318,815 PubMed articles. The number of annotations corresponding to each annotation type is shown in Table 1.

**Table 1:** PubTator annotation results.

**Annotation types**	**Counts**
Disease	1,051,402
Gene	242,436
Chemical	177,226
Mutation	100,879
Species	57,985
Cell line	4,148
Genus	255
Strain	56

#### OMIM category

We categorized 45,484 references from OMIM into 18 different OMIM groups (i.e., sections), as shown in Table 2. Notably, one article might be grouped into multiple categories and multiple articles can be grouped into one same category for one RD. For instance, there are 15 articles associated with one OMIM concept (OMIM:200110, ABLEPHARON-MACROSTOMIA SYNDROME) and 12 of them are talking about clinical features for this disease, which can be viewed by executing the Cypher Query 3.

**Table 2:** Results of OMIM category.

**OMIM categories**	**#Articles**
Clinical features	20,406
Molecular genetics	11,959
See also	4,732
Mapping	4,065
Inheritance	3,312
Description	3,114
Animal model	1,775
Pathogenesis	1,640
Clinical management	1,392
Cytogenetics	1,303
Population genetics	1,240
Diagnosis	1,190
History	1,119
Genotype phenotype correlations	737
Biochemical features	717
Other features	525
Nomenclature	359
Heterogeneity	246

Cypher Query 3.

MATCH p =(a:Article)-[:HAS_OMIM_REF]-(o:OMIMRef)
WHERE o.omimId = ′OMIM:200110′
RETURN  DISTINCT  a.pubmed_id  AS  PubMed_ID,
o.omimSections AS OMIM_Category

#### Epidemiology study tag

ES-Predict has been applied on 1,362,819 PubMed articles and it resulted in 254,723 articles being tagged as epidemiology study (“isEpi=Y”), which is accessible *via* the Cypher Query 4.

Cypher Query 4.

MATCH p = (a:Article) WHERE a.isEpi = ′Y′ RETURN
DISTINCT a.pubmed_id

### Results of the KG

The KG includes 9,643,202 nodes and 46,525,347 edges. Table 3 shows the number of nodes associated with each primary class. There are two types of FullTextUrl provided for most of the articles, i.e., Url to its full text in PDF, or Url to its journal page, so that the number of FullTextUrl is about two times more than the number of articles.

**Table 3:** Statistical results for the KG.

**Primary classes**	**#Nodes**
Author	3,154,451
FullTextUrl	2,264,836
Substance	47,506
PubtatorAnnotation	1,634,387
Article	1,362,819
Keyword	556,496
JournalVolume	537,679
MeshTerm	46,862
OMIMRef	18,455
Journal	13,485
Disease	6,061
MeshQualifier	165

## Case studies

To demonstrate the use of this graph for supporting RD research, we performed two case studies.

### Case study 1: Rare disease research landscape assessment

Ehlers-Danlos syndromes (EDS; GARD:0006322) is a heterogeneous group of diseases characterized by fragility of the soft connective tissues resulting in widespread skin, ligament, joint, blood vessel, and/or internal organ manifestations^9^. To overview its research landscape, we executed the Cypher Query 5 and obtained 2,272 PubMed articles associated with thirteen types of EDS shown in Table 4. It is noteworthy that 384 (17%) of those retrieved articles are predicted as epidemiology studies with the tag of “isEpi = Y.”

**Table 4:** Thirteen types of “Ehlers-Danlos syndrome”.

**GARD ID**	**GARD Name**
GARD:0002081	Hypermobile Ehlers-Danlos syndrome
GARD:0002082	Vascular Ehlers-Danlos syndrome
GARD:0002083	Kyphoscoliotic Ehlers-Danlos syndrome
GARD:0002084	Arthrochalasia Ehlers-Danlos syndrome
GARD:0002088	Classical Ehlers-Danlos syndrome
GARD:0002089	Dermatosparaxis Ehlers-Danlos syndrome
GARD:0006322	Ehlers-Danlos syndromes
GARD:0008486	Musculocontractural Ehlers-Danlos syndrome
GARD:0008507	Classical-like Ehlers-Danlos syndrome
GARD:0008508	Ehlers-Danlos syndrome, dysfibronectinemic type
GARD:0009991	Spondylodysplastic Ehlers-Danlos syndrome
GARD:0012474	Periodontal Ehlers-Danlos syndrome
GARD:0012613	Cardiac-Valvular Ehlers-Danlos syndrome

Cypher Query 5.

MATCH p=(d:Disease)-[r:MENTIONED_IN]->(a:Article)
WHERE (d.name) CONTAINS ′Ehlers-Danlos syndrome′
RETURN   d.gard_id   AS   GARD_ID,  d.name   AS  
GARD_Name,  a.pubmed_id   AS   PubMed_ID,  a.title   AS  
Title, a.abstractText   AS   Abstract, a.firstPublicationDate   AS  
Publication_Date, a.isEpi   AS   isEpiStudy

**Figure 4:**
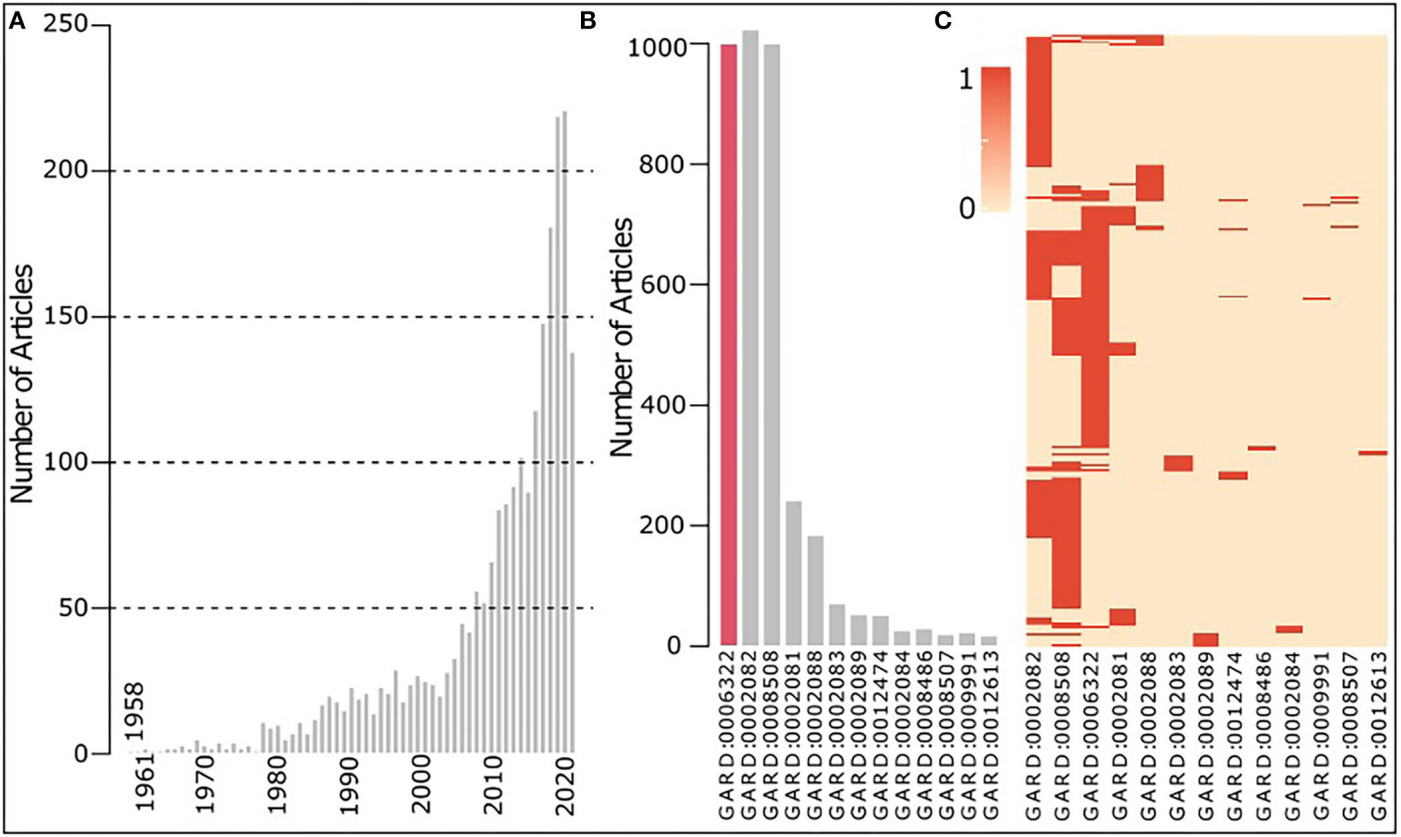
Overview of EDS research collected in PubMed between 1900 and 2021. **(A)** Research on EDS increased over years since its first publication in 1958. **(B)** Number of articles on each type of EDS with the bar of the parent EDS (GARD:0006322) in red. **(C)** Heatmap showing multiple types of EDS (column) discussed in one article (row), in dark red indicating the disease mentioned in an article.

As shown in Figure 4A, research on EDS increased significantly over years since its first publication in 1958. In Figure 4B, EDS (GARD:0006322), vascular EDS (GARD:0002082), and dysfibronectinemic EDS (GARD:0008508) have been mostly studied. Furthermore, we clustered these thirteen EDS based on their related articles by calculating their Euclidian distance, and the heatmap is shown in Figure 4C, which illustrates that published studies are largely overlapped among these three EDS. Vascular EDS is also known as Type IV EDS (EDS4), caused by a biallelic mutation in the COL3A1 gene (https://omim.org/entry/130050) which encodes for the pro-alpha1-chains of type III procollagen. Dysfibronectinemic EDS is also known as Type X EDS (EDSX), caused by a functionally abnormal fibronectin (FN1) (https://www.omim.org/entry/225310). Both COL3A1 and FN1 are genes involved in epithelial–mesenchymal transition (EMT), usually seen in embryonic development and related to cancer prognosis (Vasaikar et al., 2021), which might be the reason they always have been investigated together.

### Case study 2: Application of drug repurposing

**Figure 5:**
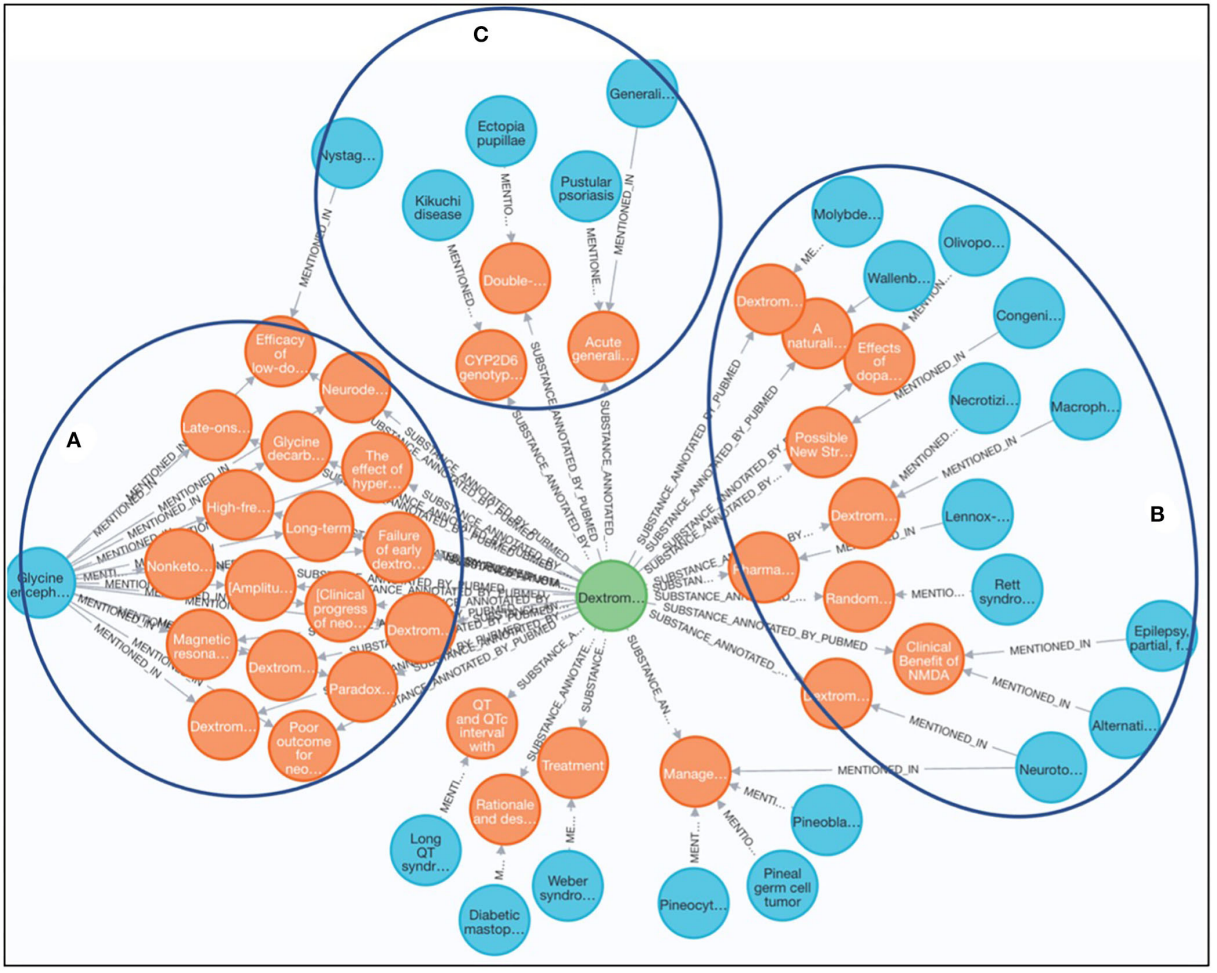
Discovery of alternative use of dextromethorphan for other rare diseases. **(A)** Dextromethorphan was widely studied for glycine encephalopathy (GE, GARD:0007219). **(B)** Dextromethorphan has potential usage for seven rare neurological diseases including Rett syndrome. **(C)** Five RDs including Ectopia pupillae and Pustular psoriasis have been studied as side effects of Dextromethorpha.

Nuedexta (DEXTROMETHORPHAN HYDROBROMIDE) is an oral medication approved by the U.S. Food and Drug Administration (FDA) in 2010 to treat pseudobulbar affect (PBA), a condition characterized by sudden and unpredictable episodes of crying or laughing seen in people with amyotrophic lateral sclerosis (ALS) and other neurological conditions (Sever et al., 2022)^10^. In this case study, we aimed at investigating the potential use of Dextromethorphan for other RDs as an application of drug repurposing. As shown in Figure 5, three main clusters were manually identified by reviewing the results from the Cypher Query 6 to discover any RDs potentially associated with Dextromethorphan as a substance reported in the articles. For the cluster A, it shows Dextromethorphan was widely studied for glycine encephalopathy (GE, GARD:0007219), an inherited metabolic disease characterized by abnormally high levels of an amino acid called glycine, which is a chemical messenger that transmits signals in the brain. Among the seventeen articles discussing Dextromethorphan and GE, 15 of them investigated Dextromethorphan as one potential therapeutic usage for GE in infants, one subtype of GE, which might be owing to the shared clinical features including intellectual disability, abnormal movements, and behavioral problems between GE and ALS. For the cluster B, it shows Dextromethorphan has potential usage for seven rare neurological diseases including Rett syndrome, Macrophage activation syndrome, Lennox-Gastaut syndrome, and Molybdenum cofactor deficiency. Glycine level might be affected by Dextromethorphan treatment for these RDs, for example, through methylation-related mechanism in Rett syndrome (with a mutation in methyl-CpG-binding protein 2 [MECP2]) or through glycogen synthase kinase in Macrophage activation syndrome (Chen et al., 2018). For the cluster C, five RDs including Ectopia pupillae and Pustular psoriasis have been studied as side effects of Dextromethorphan, which provides interesting hints to support drug repurposing *via* side effects offering a human phenotypic profile for the drug, and this profile might suggest additional disease indications (Yang and Agarwal, 2011). It is worthy to note that we manually reviewed and clustered the results in the aforementioned three groups and, additionally, we identified four ungrouped articles are false positives, which are not correlated to Dextromethorphan. The complete review results can be found in Supplementary material.

Cypher Query 6:

MATCH P = (d:Disease)-[:MENTIONED_IN]-(a:Article)-
[:SUBSTANCE_ANNOTATED_BY_PUBMED]-
(s:Substance)
WHERE toLower (s.name) STARTS WITH ‘dextrome'
RETURN P

## Discussion

In this study, we introduced a scientific annotation-based KG derived from PubMed to support RD-related research. This KG contains and represents RD-related scientific articles and their associated annotations in a semantic form, which effectively supports the research landscape assessment and research discovery in RD.

In this preliminary study, a maximum of 1,000 PubMed articles were retrieved for analysis. We anticipate false positives might exist in PubMed search results. For example, “bowel syndrome” as a search term is translated by PubMed as (“intestines”[MeSH Terms] OR “intestines”[All Fields] OR “bowel”[All Fields]) AND (“syndrome“[MeSH Terms] OR “syndrome”[All Fields]) to search-related PubMed articles. In this case, if articles have mentioned “intestines” or “bowel” and “syndrome,” instead of “bowel syndrome” as a single term, they are returned. In this preliminary study, we did not perform a manual examination among the retrieved PubMed articles. However, we created three data properties attached to the primary class (i.e., node) of “Article,” namely “pubmed_evidence” and “omim_evidence” to indicate the source of articles, and “refInOMIM” to indicate the article is from PubMed with “pubmed_evidence=true,” but also referenced in OMIM (“refInOMIM=true”). These three data properties add different weights to the articles, particularly more weight to those references from OMIM.

We explored PubTator to annotate our collected PubMed titles and abstracts and integrated those annotations in our KG for supporting research. PubTator annotates eight different types of biomedical concepts from PubMed articles, and those annotations, as the main types of concepts in the biomedical field, have a full capacity of supporting scientific research. For case study 2, we can recompose the Cypher Query 6 by exploring annotations from PubTator instead, shown as the Cypher Query 7, and more potential connections between RD and Dextromethorphan can be generated for supporting drug repurposing. Notably, multiple PubatorAnnotation nodes with the infons_type of “Chemical” referring to Dextromethorphan are associated with the same MeSH ID of MeSH:D003915, which is because we kept original annotations from titles and abstracts separately in the current version. For the next step, we will merge annotations with the same concept IDs despite their original sources and text presentations to avoid duplicates presented in the graph. Meanwhile, we acknowledged the limitation of PubTator, only working with PubMed indexed articles and annotating eight types of semantic concepts from the articles. To expand the collection of scientific annotations, we proposed several steps of extension, namely, (1) we will include more RD-related publications referenced by other resources including Orphanet and MONDO, without a restriction of PubMed indexed articles and (2) we will employ MetaMap (Aronson and Lang, 2010), an NLP tool of mapping biomedical text to the UMLS Metathesaurus, to annotate articles including non-PubMed indexed articles with additional types of annotations generated based on the UMLS semantic types.

Cypher Query 7.

MATCH P = (d:Disease)-[:MENTIONED_IN]-(a:Article)-
[:ANNOTATION_FOR]-(s:PubtatorAnnotation)
WHERE toLower (s.text) STARTS WITH ′dextrome′
RETURN P

Besides semantic annotations we gathered from PubTator, we integrated additional types of annotations to better organize the collected PubMed articles. We clustered articles based on OMIM profiles, which illustrates how OMIM references those articles to curate their disease-related content accordingly. For instance, we can easily identify five papers describing molecular genetics for EDS by executing the Cypher Query 8. To expand the article categories from this study, we propose to apply categorized articles from OMIM as training data to develop a predictive model to further cluster the remaining articles collected from this study. Furthermore, given the importance of epidemiology study in the RD field, we defined a data property of “isEpi” attached to the article node to indicate whether this article is epidemiology study related, by implementing our previously developed an LSTM-based RNN model for epidemiology study prediction. One example is shown in Case study 1 to retrieve epidemiology studies for EDS. To enrich this KG with more annotations, we proposed to integrate epidemiological information extracted from those epidemiology studies and a natural history study indicator, which will be described in a separate manuscript.

Cypher Query 8.

MATCH p = (d:Disease)-[:MENTIONED_IN]-(a:Article)-
[:HAS_OMIM_REF]-(o:OMIMRef)
where   d.gard_id  =  ′GARD:   0006322′ and any(x in
o.omimSections where x =′molecularGenetics′)
return p

The scientific evidence-based KG introduced in this article provides an important tool/resource to overview the RD research landscape. It can also serve as a data foundation for supporting various RD-related application developments, including novel associations between drugs and diseases for drug repurposing, which are shown in the “Case studies” and the “Discussion” sections. Since authors and their affiliations are included in this KG, a collaborative network can be established among authors who are co-authors in the same articles or share similar research interests based on PubTator annotations. As a scientific evidence repository, it supports multi-KG integration with the NGKG (Zhu et al., 2020) (https://disease.ncats.io/browser/) and the grant-based KG (http://grants4rd.ncats.io:7474/browser/) (Zhu et al., 2021) based on their shared nodes including Disease nodes (i.e., GARD diseases) and Article nodes (i.e., PMIDs) for scientific evidence-based research study in RD.

## Data availability statement

The original contributions presented in the study are included in the article/Supplementary material, further inquiries can be directed to the corresponding author/s.

## Author contributions

QZ conceived and supervised the project and drafted the manuscript. CQ managed the project and helped with the case studies. RL conducted data analysis and developed the KG. GV and AC participated in the initial design/development of this project, while they worked at HHS. Ð-TN helped on Neo4j graph database setup and participated in the discussion, while he worked at NCATS. ES, EM, and YX participated in the discussion. All authors read and approved the manuscript.

## Funding

This research was supported in part by the Intramural (ZIA TR000417-03) and Extramural research program of the NCATS, NIH. GV's and AC's study was supported *via* HHS Data Science CoLab, from the HHS Office of Business Management and Transformation, and the HHS Office of the Chief Technology Officer.

## Conflict of interest

GV worked at HHS and is employed by GMG ArcData, LLC. AC worked at HHS and is employed by Data Decode, LLC. Ð-TN worked at NCATS and is employed by Pfizer. The remaining authors declare that the research was conducted in the absence of any commercial or financial relationships that could be construed as a potential conflict of interest.

## Publisher's note

All claims expressed in this article are solely those of the authors and do not necessarily represent those of their affiliated organizations, or those of the publisher, the editors and the reviewers. Any product that may be evaluated in this article, or claim that may be made by its manufacturer, is not guaranteed or endorsed by the publisher.

## Footnotes

^1^*Get the Facts on Rare Diseases*. Available online at: https://www.raregenomics.org/rare-disease-facts (accessed April 3, 2022).

^2^*Neo4j*. Available online at: https://neo4j.com/ (accessed April 3, 2022).

^3^*Genetic and Rare Diseases (GARD)*. Available online at: https://rarediseases.info.nih.gov/ (accessed April 3, 2022).

^4^*Medical Subject Headings*. Available online at: https://www.nlm.nih.gov/mesh/meshhome.html (accessed April 4, 2022).

^5^*Substance Name (NM) - MEDLINE*^®^*/PubMed*^®^ *Data Element (Field) Descriptions*. Available online at: https://www.nlm.nih.gov/bsd/mms/medlineelements.html#nm (accessed October 4, 2021).

^6^*Europe PMC Articles RESTful API*. Available online at: https://europepmc.org/RestfulWebService (accessed April 4, 2022).

^7^*OMIM API*. Available online at: https://www.omim.org/api (accessed October 3, 2021).

^8^*PubTator API*. Available online at: https://www.ncbi.nlm.nih.gov/research/pubtator/api.html (accessed March 9, 2022).

^9^*Ehlers Danlos Syndromes*. Available online at: https://rarediseases.info.nih.gov/diseases/6322/ehlers-danlos-syndromes (accessed March 21, 2022).

^10^*Nuedexta*. Available online at: https://alsnewstoday.com/nuedexta/#:~:text=Nuedexta%20is%20an%20oral%20medication,ALS)%20and%20other%20neurological%20conditions. (accessed March 30, 2022).
