# Supplementary material for: Rare disease-based scientific annotation knowledge graph
Source: Front Artif Intell. 2022 Aug 11;5:932665. doi: 10.3389/frai.2022.932665 (PMC9403737; doi:10.3389/frai.2022.932665)
Supplement: Supplementary file 1 [file Data_Sheet_1.PDF]

Cypher Query 0.

```
MATCH p = (g:Disease)-[:MENTIONED_IN]-(a:Article)-[]-(n) WHERE g.name =
'Adrenomyodystrophy' RETURN p
```

Cypher Query 1.

```
MATCH p = (d:DATA)-[:PAYLOAD]->(g:S_GARD) WHERE d.is_rare = True
RETURN DISTINCT d.gard_id AS GARD_ID, d.name AS GARD_Name
```

Cypher Query 2.

```
MATCH (o:S_ORDO_ORPHANET)-[:R_exactMatch|:R_equivalentClass]-(m:S_MONDO)-
[:R_exactMatch|:R_equivalentClass]-(n:S_GARD)<-[:PAYLOAD]-(d:DATA)
WHERE d.is_rare=true  WITH o,n,m,d
MATCH (o)-[e:R_exactMatch|R_closeMatch]-(k:S_OMIM)<-[:PAYLOAD]-(h:DATA)
RETURN DISTINCT d.gard_id as GARD_ID, d.name as GARD_Name, e.name as
Orphanet_Match_Type, h.notation as OMIM_ID, h.label as OMIM_Name
ORDER BY GARD_ID
```

Cypher Query 3.

```
MATCH p =(a:Article)-[:HAS_OMIM_REF]-(o:OMIMRef) WHERE o.omimId = 'OMIM:200110'
RETURN DISTINCT a.pubmed_id AS PubMed_ID, o.omimSections AS OMIM_Category
```

Cypher Query 4.

```
MATCH p = (a:Article) WHERE a.isEpi = 'Y' RETURN DISTINCT a.pubmed_id
```

Cypher Query 5.

```
MATCH p=(d:Disease)-[r:MENTIONED_IN]->(a:Article)
WHERE (d.name) CONTAINS 'Ehlers-Danlos syndrome'
RETURN d.gard_id AS GARD_ID, d.name AS GARD_Name, a.pubmed_id AS PubMed_ID, a.title
AS Title, a.abstractText AS Abstract, a.firstPublicationDate AS Publication_Date, a.isEpi AS
isEpiStudy
```

Cypher Query 6:

```
MATCH P = (d:Disease)-[:MENTIONED_IN]-(a:Article)-[:SUBSTANCE_ANNOTATED_BY_PUBMED]-
(s:Substance)
WHERE toLower (s.name) STARTS WITH ‘dextrome’
RETURN P
```

Cypher Query 7.

```
MATCH P = (d:Disease)-[:MENTIONED_IN]-(a:Article)-[:ANNOTATION_FOR]-
(s:PubtatorAnnotation)
WHERE toLower (s.text) STARTS WITH 'dextrome'
```

```
RETURN P
```

<u>Cypher Query 8.</u>

```
MATCH p = (d:Disease)-[:MENTIONED_IN]-(a:Article)-[:HAS_OMIM_REF]-(o:OMIMRef) where
d.gard_id = 'GARD: 0006322' and any(x in o.omimSections where x ='molecularGenetics') return
p
```
